# Redetermination of brackebuschite, Pb_2_Mn^3+^(VO_4_)_2_(OH)

**DOI:** 10.1107/S2056989016001948

**Published:** 2016-02-06

**Authors:** Barbara Lafuente, Robert T. Downs

**Affiliations:** a University of Arizona, 1040 E. 4th Street, Tucson, AZ 85721-0077, USA

**Keywords:** crystal structure, redetermination, brackebuschite, Raman spectroscopy

## Abstract

The crystal structure of brackebuschite, ideally Pb_2_Mn^3+^(VO_4_)_2_(OH) [dilead(II) manganese(III) vanadate(V) hydroxide], was redetermined based on single-crystal X-ray diffraction data of a natural sample from the type locality Sierra de Cordoba, Argentina. Improving on previous results, anisotropic displacement parameters for all non-H atoms were refined and the H atom located, obtaining a significant improvement of accuracy and an unambiguous hydrogen-bonding scheme. Brackebuschite belongs to the brackebuschite group of minerals with general formula *A*_2_ *M*(*T*1O_4_)(*T*2O_4_)(OH, H_2_O), with *A* = Pb^2+^, Ba, Ca, Sr; *M* = Cu^2+^, Zn, Fe^2+^, Fe^3+^, Mn^3+^, Al; *T*1 = As^5+^, P, V^5+^; and *T*2 = As^5+^, P, V^5+^, S^6+^. The crystal structure of brackebuschite is based on a cubic closest-packed array of O and Pb atoms with infinite chains of edge-sharing [Mn^3+^O_6_] octa­hedra located about inversion centres and decorated by two unique VO_4_ tetra­hedra (each located on a special position 2*e*, site symmetry *m*). One type of VO_4_ tetra­hedra is linked with the ^1^_∞_[MnO_4/2_O_2/1_] chain by one common vertex, alternating with H atoms along the chain, while the other type of VO_4_ tetra­hedra link two adjacent octa­hedra by sharing two vertices with them and thereby participating in the formation of a three-membered Mn_2_V ring between the central atoms. The ^1^_∞_[Mn^3+^(VO_4_)_2_OH] chains run parallel to [010] and are held together by two types of irregular [PbO_*x*_] polyhedra (*x* = 8, 11), both located on special position 2*e* (site symmetry *m*). The magnitude of the libration component of the O atoms of the ^1^_∞_[Mn^3+^(VO_4_)_2_OH] chain increases linearly with the distance from the centerline of the chain, indicating a significant twisting to and fro of the chain along [010]. The hy­droxy group bridges one Pb^2+^ cation with two Mn^3+^ cations and forms an almost linear hydrogen bond with a vanadate group of a neighbouring chain. The O⋯O distance of this inter­action determined from the structure refinement agrees well with Raman spectroscopic data.

## Abstract 2

The crystal structure of brackebuschite, ideally Pb_2_Mn^3+^(VO_4_)_2_(OH), was redetermined based on single-crystal X-ray diffraction data of a natural sample from the type locality Sierra de Cordoba, Argentina. Improving on previous results, anisotropic displacement parameters for all non-H atoms were refined and the H atom located, obtaining a significant lowering of the reliability factors.

## Mineralogical and crystal-chemical context   

Brackebuschite, ideally Pb_2_Mn^3+^(VO_4_)_2_(OH), belongs to the brackebuschite group of minerals with monoclinic symmetry and space group type *P*2_1_/*m*. The brackebuschite group is defined by the general formula *A*_2_ *M*(*T*1O_4_)(*T*2O_4_)(OH, H_2_O), with *A* = Pb^2+^, Ba, Ca, Sr; *M* = Cu^2+^, Zn, Fe^2+^, Fe^3+^, Mn^3+^, Al; *T*1 = As^5+^, P, V^5+^; and *T*2 = As^5+^, P, V^5+^, S^6+^. Together with brackebuschite, other secondary lead minerals within this group include arsenbrackebuschite [Pb_2_(Fe^3+^,Zn)(AsO_4_)_2_(OH,H_2_O)] (Abraham *et al.*, 1978 ▸), calderónite [Pb_2_Fe^3+^(VO_4_)_2_(OH)] (González del Tánago *et al.*, 2003 ▸), tsumebite [Pb_2_Cu(PO_4_)(SO_4_)(OH)] (Nichols, 1966 ▸), arsen­tsumebite [Pb_2_Cu(AsO_4_)(SO_4_)(OH)] (Bideaux *et al.*, 1966 ▸; Zubkova *et al.*, 2002 ▸), bushmakinite [Pb_2_Al(PO_4_)(VO_4_)(OH)] (Pekov *et al.*, 2002 ▸), ferribushmakinite [Pb_2_Fe^3+^(PO_4_)(VO_4_)(OH)] (Kampf *et al.*, 2015 ▸), feinglosite [Pb_2_Zn(AsO_4_)_2_·H_2_O] (Clark *et al.*, 1997 ▸), and possibly heyite [Pb_5_Fe^2+^_2_O_4_(VO_4_)_2_], in which a cursory X-ray diffraction investigation suggest a similarity with brackebuschite (Williams, 1973 ▸).

Other lead minerals related to the brackebuschite group include fornacite [CuPb_2_(CrO_4_)(AsO_4_)(OH)] (Cocco *et al.*, 1967 ▸; Fanfani & Zanazzi, 1967 ▸), molybdofornacite [CuPb_2_(MoO_4_)(AsO_4_)(OH)] (Medenbach *et al.*, 1983 ▸), and vauque­len­ite [CuPb_2_(CrO_4_)(PO_4_)(OH)] (Fanfani & Zanazzi, 1968 ▸). These minerals demonstrate a richness to the crystallography of the group because the first two are described in space group *P*2_1_/*c* with doubled *c*-cell edge, while the last one is described in *P*2_1_/*n* and exhibits doubling of both the *a*- and *c*-cell edges (Fanfani & Zanazzi, 1967 ▸).

In addition to the lead minerals, the brackebuschite group of minerals also includes bearthite [Ca_2_Al(PO_4_)_2_OH] (Chopin *et al.*, 1993 ▸), canosioite [Ba_2_Fe^3+^(AsO_4_)_2_(OH)] (Hålenius *et al.*, 2015 ▸), gamagarite [Ba_2_Fe^3+^(VO_4_)_2_(OH)] (de Villiers *et al.*, 1943 ▸; Basso *et al.*, 1987 ▸), tokyoite [Ba_2_Mn^3+^(VO_4_)_2_(OH)] (Matsubara *et al.*, 2004 ▸), goedkenite [Sr_2_Al(PO_4_)_2_(OH)] (Moore *et al.*, 1975 ▸), and grandaite [Sr_2_Al(AsO_4_)_2_(OH)] (Cámara *et al.*, 2014 ▸).

**Figure 1:**
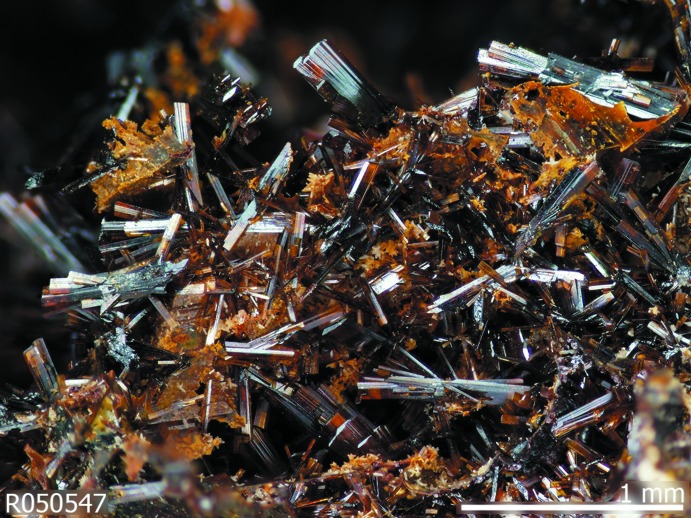
Photograph of the brackebuschite specimen analysed in this study.

In the course of characterizing minerals for the RRUFF Project (http://rruff.info), we were able to isolate a single crystal of brackebuschite from the type locality Sierra de Cordoba (Argentina), with composition Pb_1.91_(Mn^3+^_0.81_Fe^3+^_0.07_Zn_0.07_)_Σ=0.95_(V_1.98_As_0.02_)_Σ=2.00_O_8.00_(OH)_1.00_. The ratio Mn:Fe varies from grain to grain as shown in Fig. 1 ▸, where the colour of the crystals range from light-brown (Mn-rich, this crystal) to dark-brown [Fe-rich, (Mn^3+^_0.43_Fe^3+^_0.42_Zn_0.10_)_Σ=0.95]_. González del Tánago *et al.* (2003 ▸) suggested that brackebuschite [Pb_2_Mn^3+^(VO_4_)_2_(OH)] and calderónite [Pb_2_Fe^3+^(VO_4_)_2_(OH)] probably form a complete solid solution, as both phases have been found to coexist on a single specimen.

The crystal structure of brackebuschite was first determined by Donaldson & Barnes (1955 ▸) in space group *B*2_1_/*m* assuming a chemical formula Pb_2_Mn^2+^(VO_4_)_2_·H_2_O. Foley *et al.* (1997 ▸) redefined its structure in space group *P*2_1_/*m* and revised its composition to the currently accepted Pb_2_Mn^3+^(VO_4_)_2_(OH). Structure refinement of the latter converged at a reliability factor *R*1 of 0.056 and was based on anisotropic displacement parameters for all non-O atoms [note that the deposited data in the Inorganic Crystal Structure Database (ICSD, 2016 ▸), entry #89256, report only isotropic displacement parameters], and the H atom undetermined. In the current work, all non-hydrogen atoms were refined with anisotropic displacement parameters, and the H atom was located, leading to a significant improvement of accuracy and precision, and to an unambiguous hydrogen bonding scheme.

## Structural commentary   

**Figure 2:**
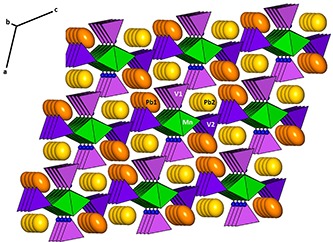
Crystal structure of brackebuschite. The edge-sharing [MnO_6_] octa­hedra (green) form chains parallel to [010] with V1O_4_ and V2O_4_ tetra­hedra (purple and violet, respectively) linked to them. These chains are held together by Pb1 and Pb2 (orange and yellow ellipsoids, respectively). Blue spheres of arbitrary radius represent H atoms.

The structure of brackebuschite is characterized by a distorted cubic closest-packed array of O and Pb atoms along [10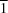] as stacking direction. Infinite chains of edge-sharing [Mn^3+^O_6_] octa­hedra decorated by V1O_4_ and V2O_4_ tetra­hedra are aligned parallel to [010], perpendicular to the stacking direction. Mn^3+^, located on an inversion centre, is coordinated by the oxygen anions belonging to VO_4_ tetra­hedra (O3×2 and O5×2) and the hydroxyl group (O7×2) in an overall distorted octa­hedral arrangement. Each V1O_4_ tetra­hedron is linked to the ^1^_∞_[MnO_4/2_O_2/1_] chain by one common vertex (O3) while each V2O_4_ links two adjacent octa­hedra by sharing two vertices (O5) with them. The V1O_4_ groups and the H atoms alternate, belong to the edge-sharing atoms and are arranged along one side of the ^1^_∞_[MnO_4/2_O_2/1_] chain. The thus resulting ^1^_∞_[Mn^3+^(VO_4_)_2_OH] chains are held together by irregular [Pb1O_11_] and [Pb2O_8_] polyhedra (Fig. 2 ▸).

**Figure 3:**
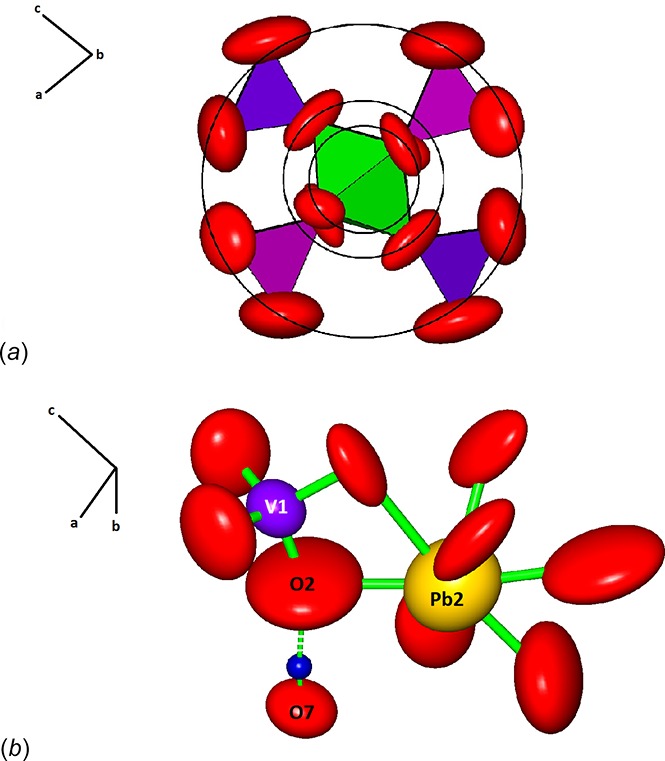
(*a*) View of the ^1^_∞_[Mn^3+^(VO_4_)_2_OH] chain looking down [010]. The black rings represent different radii that correlate with the progressive increase of the libration component of oxygen atoms further away from the centre of the chain. Purple and violet tetra­hedra and green octa­hedra represent V1O_4_, V2O_4_ and [MnO_6_], respectively. Red ellipsoids represent O atoms; (*b*) large O2 disk-shaped ellipsoid oriented perpendicular to the V1—O bond (anisotropic displacement ellipsoids at the 99% probability level). Note the hydrogen bond O2⋯H—O7 (dashed lines). Purple, yellow and red ellipsoids represent V1O_4_, [Pb2O_8_] and O atoms, respectively. The blue sphere represents the H atom.

Looking down the axis of the [Mn^3+^(VO_4_)_2_OH] chain, there is a radial increase in the amplitude of the displacement parameters. We inter­pret this to indicate that the entire chain is undergoing rigid-body libration, oscillating to and fro along its axis. The radial change in amplitude is indicated by three concentric rings in Fig. 3 ▸ *a*. The average amplitudes of the inner, middle, and outer rings (1.34, 2.00, and 4.06 Å, respectively) increase roughly linearly with the radial distance from the chain axis.

**Figure 4:**
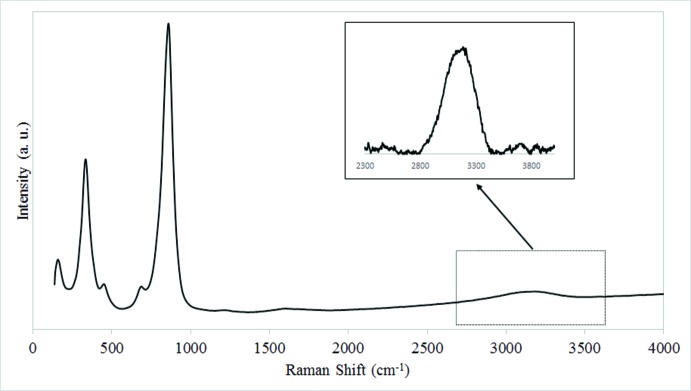
Raman spectrum of brackebuschite. The weak Raman band around 3145 cm^−1^ is assigned to the OH stretching vibrations associated with the OH group (ν_OH_).

Bond-valence calculations (Brown, 2002 ▸) confirm that O7 corresponds to the hydroxyl group (bond-valence sum of 1.25 valence units), which is approximately tetra­hedrally coordinated by three cations and O2 (bond-valence sum of 1.61 v.u.) with which it forms an almost linear hydrogen bond (Table 1 ▸). The Raman spectrum of brackebuschite (Fig. 4 ▸) shows a broad band around 3145 cm^−1^ that is assigned to the OH-stretching vibration (υ_OH_). According to the correlation of O—H IR stretching frequencies and O—H⋯O hydrogen-bond lengths in minerals (Libowitzky, 1999 ▸), the stretching frequency inferred from this bond-length is 3143 cm^−1^.

The O2 atom, the one associated as the acceptor atom of the hydrogen bond, displays quite large anisotropic displacement parameters relative to the other atoms (Fig. 3 ▸ *b*). The disk-shaped ellipsoid is oriented parallel to the weaker Pb2—O bond and perpendicular to the stronger V1—O bond, which constrains the thermal vibration in that direction.

## Synthesis and crystallization   

The natural brackebuschite specimen used in this study is from the type locality Sierra de Cordoba, Argentina, and is in the collection of the RRUFF project (http://rruff.info/R050547). The chemical composition of the brackebuschite specimen was determined with a CAMECA SX100 electron microprobe operated at 20 kV and 20 nA. Seven analysis points yielded an average composition (wt. %): PbO 60.8 (4), V_2_O_5_ 25.6 (2), Mn_2_O_3_ 9.1 (5), Fe_2_O_3_ 0.8 (5), ZnO 0.8 (2), and As_2_O_5_ 0.33 (8), with H_2_O 1.28 estimated to provide one H_2_O mol­ecule per formula unit. The empirical chemical formula, based on nine oxygen atoms, is Pb_1.91_(Mn^3+^_0.81_Fe^3+^_0.07_Zn_0.07_)_Σ=0.95_(V_1.98_As_0.02_)_Σ=2.00_O_8.00_(OH)_1.00_.

## Refinement   

Crystal data, data collection and structure refinement details are summarized in Table 2 ▸. Electron microprobe analysis revealed that the brackebuschite sample studied here contains small amounts of Fe, Zn and As. However, the structure refinements, with and without a minor contribution of these elements in the octa­hedral and tetra­hedral sites, did not produce any significant differences in terms of reliability factors or displacement parameters. Hence, the ideal chemical formula Pb_2_Mn^3+^(VO_4_)_2_(OH) was assumed during the refinement. The H atom was located from difference Fourier syntheses and its position refined with a fixed isotropic displacement parameter (*U*_iso_ = 0.03), and soft DFIX constraint of 0.9 Å from O7. The maximum residual electron density in the difference Fourier map, 2.66 e Å^−3^, was located at (0.6661, 0.1681, 0.5521), 0.66 Å from Pb1 and the minimum, −2.16 e Å^−3^, at (0.7036, 0.25, 0.5714), 0.41 Å from Pb1.

## Supplementary Material

Crystal structure: contains datablock(s) I. DOI: 10.1107/S2056989016001948/wm5265sup1.cif

Structure factors: contains datablock(s) I. DOI: 10.1107/S2056989016001948/wm5265Isup2.hkl

CCDC reference: 1451240

Additional supporting information:  crystallographic information; 3D view; checkCIF report

## Figures and Tables

**Table 1:** Hydrogen-bond geometry (Å, °)

*D*—H⋯*A*	*D*—H	H⋯*A*	*D*⋯*A*	*D*—H⋯*A*
O7—H⋯O2^i^	0.89 (2)	1.80 (2)	2.686 (7)	174 (10)

Symmetry code: (i) 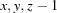.

**Table 2:** Experimental details

Crystal data	
Chemical formula	Pb_2_Mn(VO_4_)_2_(OH)
*M*_r_	716.21
Crystal system, space group	Monoclinic, *P*2_1_/*m*
Temperature (K)	293
*a*, *b*, *c* (Å)	7.6492 (1), 6.1262 (1), 8.9241 (2)
β (°)	112.195 (1)
*V* (Å^3^)	387.20 (1)
*Z*	2
Radiation type	Mo *K*α
μ (mm^−1^)	47.27
Crystal size (mm)	0.05 × 0.05 × 0.05

Data collection	
Diffractometer	Bruker APEXII CCD area detector
Absorption correction	Multi-scan (*SADABS*; Bruker, 2004 ▸)
*T*_min_, *T*_max_	0.201, 0.201
No. of measured, independent and observed [*I* > 2σ(*I*)] reflections	11674, 1521, 1356
*R*_int_	0.037
(sin θ/λ)_max_ (Å^−1^)	0.759

Refinement	
*R*[*F* ^2^ > 2σ(*F* ^2^)], *wR*(*F* ^2^), *S*	0.025, 0.056, 1.05
No. of reflections	1521
No. of parameters	82
No. of restraints	1
H-atom treatment	Only H-atom coordinates refined
Δρ_max_, Δρ_min_ (e Å^−3^)	2.67, −2.16

Computer programs: *APEX2* and *SAINT* (Bruker, 2004 ▸), *SHELXL2014* (Sheldrick, 2015 ▸), *XtalDraw* (Downs & Hall-Wallace, 2003 ▸) and *publCIF* (Westrip, 2010 ▸). Coordinates taken from a previous refinement.

## supplementary crystallographic information

### Crystal data

**Table:**

Pb_2_Mn(VO_4_)_2_(OH)	*F*(000) = 616
*M**_r_* = 716.21	*D*_x_ = 6.143 Mg m^−^^3^
Monoclinic, *P*2_1_/*m*	Mo *K*α radiation, λ = 0.71073 Å
*a* = 7.6492 (1) Å	Cell parameters from 4222 reflections
*b* = 6.1262 (1) Å	θ = 2.5–32.3°
*c* = 8.9241 (2) Å	µ = 47.27 mm^−^^1^
β = 112.195 (1)°	*T* = 293 K
*V* = 387.20 (1) Å^3^	Tabular, light-brown
*Z* = 2	0.05 × 0.05 × 0.05 mm

### Data collection

**Table:**

Bruker APEXII CCD area-detector diffractometer	1356 reflections with *I* > 2σ(*I*)
Radiation source: fine-focus sealed tube	*R*_int_ = 0.037
φ and ω scan	θ_max_ = 32.6°, θ_min_ = 2.9°
Absorption correction: multi-scan (*SADABS*; Bruker, 2004)	*h* = −11→11
*T*_min_ = 0.201, *T*_max_ = 0.201	*k* = −9→9
11674 measured reflections	*l* = −13→13
1521 independent reflections	

### Refinement

**Table:**

Refinement on *F*^2^	Hydrogen site location: difference Fourier map
Least-squares matrix: full	Only H-atom coordinates refined
*R*[*F*^2^ > 2σ(*F*^2^)] = 0.025	*w* = 1/[σ^2^(*F*_o_^2^) + (0.0195*P*)^2^ + 3.6132*P*] where *P* = (*F*_o_^2^ + 2*F*_c_^2^)/3
*wR*(*F*^2^) = 0.056	(Δ/σ)_max_ < 0.001
*S* = 1.05	Δρ_max_ = 2.67 e Å^−^^3^
1521 reflections	Δρ_min_ = −2.16 e Å^−^^3^
82 parameters	Extinction correction: *SHELXL2014* (Sheldrick, 2015), Fc^*^=kFc[1+0.001xFc^2^λ^3^/sin(2θ)]^-1/4^
1 restraint	Extinction coefficient: 0.00067 (18)

### Special details

**Table:**

Geometry. All e.s.d.'s (except the e.s.d. in the dihedral angle between two l.s. planes) are estimated using the full covariance matrix. The cell e.s.d.'s are taken into account individually in the estimation of e.s.d.'s in distances, angles and torsion angles; correlations between e.s.d.'s in cell parameters are only used when they are defined by crystal symmetry. An approximate (isotropic) treatment of cell e.s.d.'s is used for estimating e.s.d.'s involving l.s. planes.

### Fractional atomic coordinates and isotropic or equivalent isotropic displacement parameters (Å^2^)

**Table:**

	*x*	*y*	*z*	*U*_iso_*/*U*_eq_	
Pb1	0.32423 (5)	−0.2500	0.39735 (4)	0.02766 (9)	
Pb2	−0.25814 (4)	−0.2500	0.25617 (4)	0.02176 (8)	
Mn	0.0000	0.0000	0.0000	0.00476 (15)	
V1	0.55901 (14)	0.7500	0.82401 (12)	0.00821 (18)	
V2	0.96053 (14)	0.7500	0.66182 (12)	0.00864 (17)	
O1	0.4927 (5)	0.9756 (6)	0.7041 (4)	0.0167 (6)	
O2	0.4546 (8)	0.7500	0.9583 (8)	0.0304 (13)	
O3	0.8084 (6)	0.7500	0.9407 (6)	0.0124 (8)	
O4	0.7301 (8)	0.7500	0.5441 (7)	0.0256 (12)	
O5	0.0115 (5)	0.9882 (5)	0.7801 (4)	0.0147 (6)	
O6	0.0767 (8)	0.7500	0.5377 (7)	0.0249 (12)	
O7	0.1837 (6)	0.7500	0.0814 (6)	0.0102 (8)	
H	0.268 (11)	0.7500	0.034 (11)	0.030*	

### Atomic displacement parameters (Å^2^)

**Table:**

	*U*^11^	*U*^22^	*U*^33^	*U*^12^	*U*^13^	*U*^23^
Pb1	0.03242 (17)	0.02685 (15)	0.03049 (18)	0.000	0.01957 (14)	0.000
Pb2	0.02570 (15)	0.01980 (13)	0.02074 (14)	0.000	0.00988 (11)	0.000
Mn	0.0067 (3)	0.0032 (3)	0.0038 (3)	0.0001 (3)	0.0013 (3)	−0.0001 (3)
V1	0.0071 (4)	0.0080 (4)	0.0093 (4)	0.000	0.0029 (3)	0.000
V2	0.0111 (4)	0.0079 (4)	0.0073 (4)	0.000	0.0039 (4)	0.000
O1	0.0161 (15)	0.0130 (14)	0.0175 (17)	0.0020 (12)	0.0023 (13)	0.0045 (12)
O2	0.023 (3)	0.048 (4)	0.029 (3)	0.000	0.019 (3)	0.000
O3	0.0056 (18)	0.0109 (18)	0.016 (2)	0.000	−0.0019 (16)	0.000
O4	0.016 (2)	0.039 (3)	0.015 (3)	0.000	−0.001 (2)	0.000
O5	0.0274 (17)	0.0111 (13)	0.0077 (14)	−0.0031 (12)	0.0089 (13)	−0.0026 (11)
O6	0.033 (3)	0.028 (3)	0.025 (3)	0.000	0.024 (2)	0.000
O7	0.0085 (18)	0.0111 (18)	0.012 (2)	0.000	0.0054 (16)	0.000

### Geometric parameters (Å, º)

**Table:**

Pb1—O1^i^	2.563 (3)	Pb2—O6^ii^	2.830 (6)
Pb1—O7^ii^	2.611 (5)	Pb2—O3^vi^	3.045 (5)
Pb1—O6^ii^	2.635 (5)	Mn—O5^iv^	1.999 (3)
Pb1—O4^ii^	2.878 (5)	Mn—O7^vii^	2.019 (3)
Pb1—O1^ii^	2.898 (4)	Mn—O3^i^	2.046 (3)
Pb1—O5^iii^	2.933 (4)	V1—O2	1.673 (6)
Pb1—O4^i^	3.1610 (15)	V1—O1	1.703 (3)
Pb2—O1^iii^	2.579 (3)	V1—O3	1.795 (4)
Pb2—O5^iv^	2.588 (3)	V2—O6^viii^	1.661 (5)
Pb2—O4^v^	2.605 (6)	V2—O4	1.677 (5)
Pb2—O2^vi^	2.735 (6)	V2—O5^viii^	1.756 (3)
			
O5^iv^—Mn—O5^ix^	180.0	O3^i^—Mn—O3^vi^	180.0 (3)
O5^iv^—Mn—O7^vii^	92.45 (16)	O2—V1—O1	109.90 (17)
O5^ix^—Mn—O7^vii^	87.55 (16)	O1—V1—O1^x^	108.4 (3)
O5^ix^—Mn—O7^ii^	92.45 (16)	O2—V1—O3	106.0 (3)
O7^vii^—Mn—O7^ii^	180.0 (2)	O1—V1—O3	111.31 (14)
O5^iv^—Mn—O3^i^	90.67 (17)	O6^viii^—V2—O4	106.4 (3)
O5^ix^—Mn—O3^i^	89.33 (17)	O6^viii^—V2—O5^viii^	110.34 (16)
O7^vii^—Mn—O3^i^	81.87 (12)	O4—V2—O5^viii^	108.58 (16)
O7^ii^—Mn—O3^i^	98.13 (12)	O5^viii^—V2—O5^xi^	112.4 (2)
O5^iv^—Mn—O3^vi^	89.33 (17)		

Symmetry codes: (i) −*x*+1, −*y*+1, −*z*+1; (ii) *x*, *y*−1, *z*; (iii) −*x*, *y*−3/2, −*z*+1; (iv) −*x*, −*y*+1, −*z*+1; (v) *x*−1, *y*−1, *z*; (vi) *x*−1, *y*−1, *z*−1; (vii) −*x*, −*y*+1, −*z*; (viii) *x*+1, *y*, *z*; (ix) *x*, *y*−1, *z*−1; (x) *x*, −*y*+3/2, *z*; (xi) *x*+1, −*y*+3/2, *z*.

### Hydrogen-bond geometry (Å, º)

**Table:**

*D*—H···*A*	*D*—H	H···*A*	*D*···*A*	*D*—H···*A*
O7—H···O2^xii^	0.89 (2)	1.80 (2)	2.686 (7)	174 (10)

Symmetry code: (xii) *x*, *y*, *z*−1.
